# Reduced Responsiveness to Volatile Signals Creates a Modular Reward Provisioning in an Obligate Food-for-Protection Mutualism

**DOI:** 10.3389/fpls.2018.01076

**Published:** 2018-07-24

**Authors:** Omar F. Hernández-Zepeda, Rosario Razo-Belman, Martin Heil

**Affiliations:** Departamento de Ingeniería Genética, Centro de Investigación y de Estudios Avanzados del Instituto Politécnico Nacional-Irapuato, Guanajuato, Mexico

**Keywords:** exclusive rewards, extrafloral nectar, obligate mutualism, sanctions, systemic signaling, volatile signals

## Abstract

Plants in more than 100 families secrete extrafloral nectar (EFN) to establish food-for-protection mutualisms with ants. Facultative ant-plants secrete EFN as a jasmonic acid (JA)-dependent response to attract generalist ants. In contrast, obligate ant-plants like the Central American “Swollen-Thorn Acacias” are colonized by specialized ants, although an individual host can carry ant colonies from different species that differ in the degree of protection they provide. We hypothesized that hosts that associate simultaneously with various partners should produce rewards in a modular manner to preferentially reward high quality partners. To test this hypothesis, we applied JA to distinct leaves and quantified cell wall invertase activity (CWIN; a regulator of nectar secretion) and EFN secretion by these “local” (i.e., treated) and the “systemic” (i.e., non-treated) leaves of the same branch. Both CWIN activity and EFN secretion increased in local and systemic leaves of the facultative ant-plant *Acacia cochliacantha*, but only in the local leaves of the obligate ant-plant, *A. cornigera*. The systemic EFN secretion in *A. cochliacantha* was associated with an enhanced emission of volatile organic compounds (VOCs). Such VOCs function as “external signals” that control systemic defense responses in diverse plant species. Indeed, the headspace of JA-treated branches of *A. cochliacantha* induced EFN secretion in both plant species, whereas the headspace of *A. cornigera* caused no detectable induction effect. Analyses of the headspace using GC-MS identified six VOCs in the headspace of *A. cochliacantha* that were not emitted by *A. cornigera*. Among these VOCs, β-caryophyllene and (*cis*)-hexenyl isovalerate have already been reported in other plant species to induce defense traits, including EFN secretion. Our observations underline the importance of VOCs as systemic within-plant signals and show that the modular rewarding in *A. cornigera* is likely to result from a reduced emission of the systemic signal, rather than from a reduced responsiveness to the signal. We suggest that modular rewarding allows hosts to restrict the metabolic investment to specific partners and to efficiently sanction potential exploiters.

## Introduction

Most mutualisms are formed by hosts that interact with multiple partners. Partners can differ in their quality as mutualists, and non-reciprocating partners infer a cost to their host without providing the corresponding benefit (Sachs, 2015). Therefore, theory predicts the evolution of “host sanctions” or other mechanisms that allow hosts to adjust reward provisioning to the quality of the service they receive (Bshary and Grutter, 2002; Kiers et al., 2003; Kiers and Denison, 2008). Host sanctions have been reported for mutualisms such as the legume–rhizobia mutualism, in which plants were reported to “penalize” non N-fixing nodules (Kiers et al., 2003; Westhoek et al., 2017), or for the fig–fig wasp mutualism, in which the fig tree aborted figs that were colonized by non-pollinating wasps (Jandér et al., 2012). Intriguingly, Kiers et al. (2003) and Jandér et al. (2012) observed sanctions to occur in a modular manner: only nodules that did not fix nitrogen or figs that were carrying non-cooperative wasps were sanctioned. Evidently, a modular provisioning of rewards is adaptive in symbiotic systems in which different parts of the same host are colonized simultaneously by different partners. In contrast, hosts that engage in facultative mutualisms with non-symbiotic partners should provide rewards in a more systemic way, in order to enhance their attractiveness to mutualists that eventually visit the host (Agrawal and Rutter, 1998).

**Figure 1:**
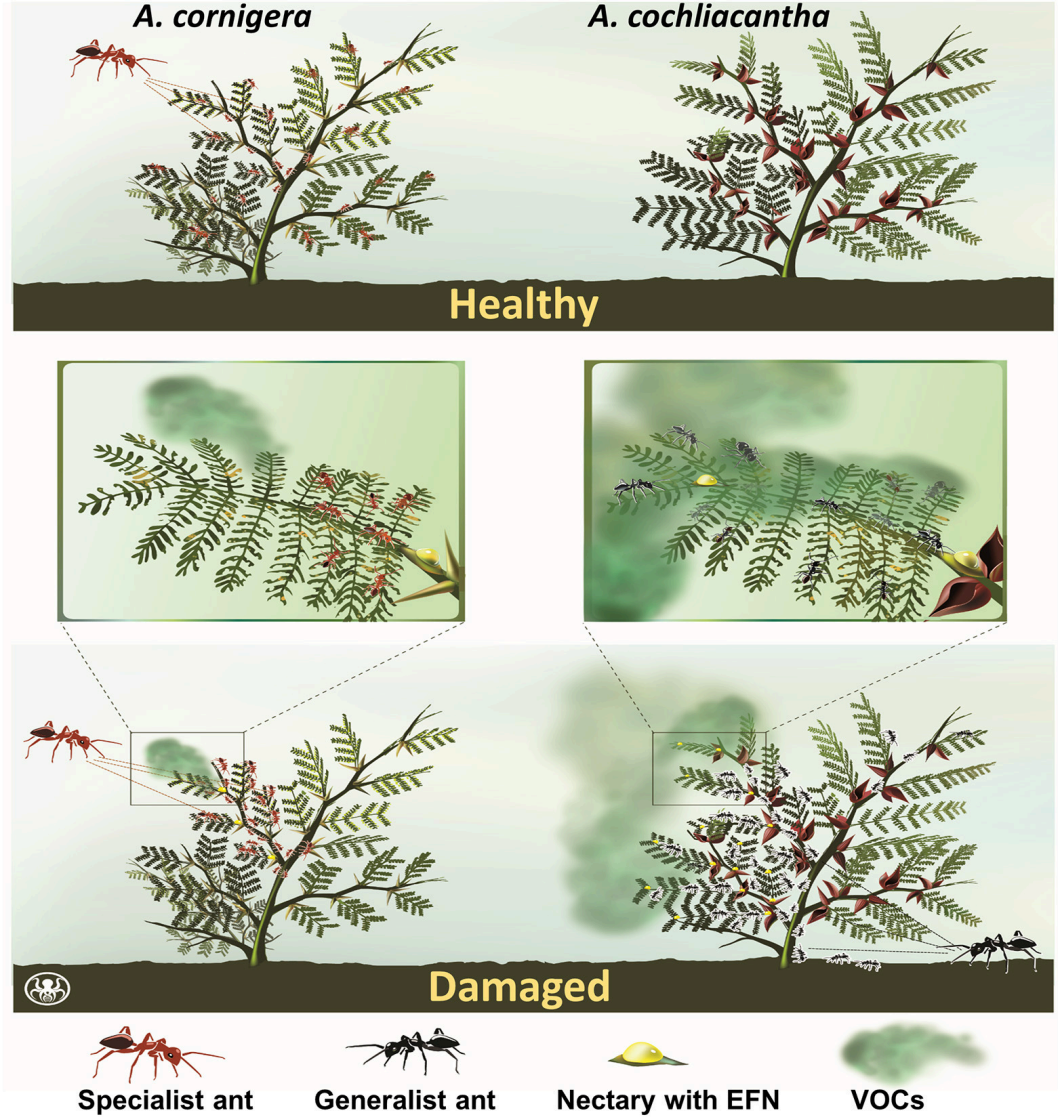
Study species and hypothesis. The obligate ant-plant, *A. cornigera* and the facultative ant-plant, *A. cochliacantha*, are hypothesized to respond differently to local damage. *A. cornigera* is colonized by a standing army of specialist ants (*P. ferrugineus*) even in the absence of damage (“healthy”, upper panel). These ants are quickly recruited when the plant is locally damaged (lower panel), a response that can be elicited by plant VOCs (Agrawal, 1998; Inui and Itioka, 2007; Mayer et al., 2008; Schatz et al., 2009) or by a strongly modular induction of extrafloral nectar (EFN) secretion (this study). In contrast, *A. cochliacantha* responds to damage with the attraction of generalist ants from the vicinity, a situation in which the number of ants recruited should correlate positively with the amount of EFN secreted. Since VOCs can control systemic plant responses (Heil and Ton, 2008), a modular versus systemic reward production by the obligate vs. the facultative ant-plant might be explained by differences in the emission of—or the response to—such VOCs.

A modular reward provisioning has been reported from symbiotic mutualisms whereas systemic reward production characterizes common facultative mutualisms, but the molecular pathways remain poorly understood that enable plants to allocate rewards in different spatial patterns. Here, we use extrafloral nectar (EFN) secretion to identify the mechanism that controls the modular *versus* a more systemic production of a reward in an ant-plant mutualism. Extrafloral nectar (EFN) is produced by plants from more than 4,000 species in ca. 750 genera (Weber and Keeler, 2013) to attract ants and other predators, or even parasitoids, all of which act as an indirect defense against herbivores (Heil, 2015). The main components of EFN are mono- and disaccharides and amino acids, but proteins are also frequently reported (Escalante-Pérez and Heil, 2012). The content of metabolically costly compounds and the observation that EFN secretion can be limited by light availability (Bixenmann et al., 2011; Jones and Koptur, 2015) indicate that EFN is a costly reward whose production should be under strict control by the plant. Most EFN-secreting plant species function as facultative ant plants, i.e., they secrete EFN in response to herbivory as a jasmonic acid (JA)-dependent defense mechanism to establish facultative mutualisms with generalist ants, which are attracted from the vicinity (Heil et al., 2001; Roy et al., 2017). The inducibility of EFN secretion by JA finds its mechanistic explanation in the fact that cell-wall invertase (CWIN), which represents a central limiting step in the secretion of nectar (Ruhlmann et al., 2010; Lin et al., 2014; Roy et al., 2017), is induced by JA (Millán-Cañongo et al., 2014). In contrast, so-called myrmecophytes, which have been described from over 100 genera of plants, provide nesting space–and usually also a food reward such as EFN - to colonies of symbiotic “plant-ants”. These interactions are considered obligate mutualisms, because the plant-ants depend on their host for food and nesting space, whereas the plants depend on the ants for protection (Heil and McKey, 2003). In simple words, facultative ant-plants recruit generalist ants from the vicinity when defense is actually required, whereas obligate ant-plants provide nesting space and food to a “standing army” of specialized ants (Figure 1).

**Figure 2:**
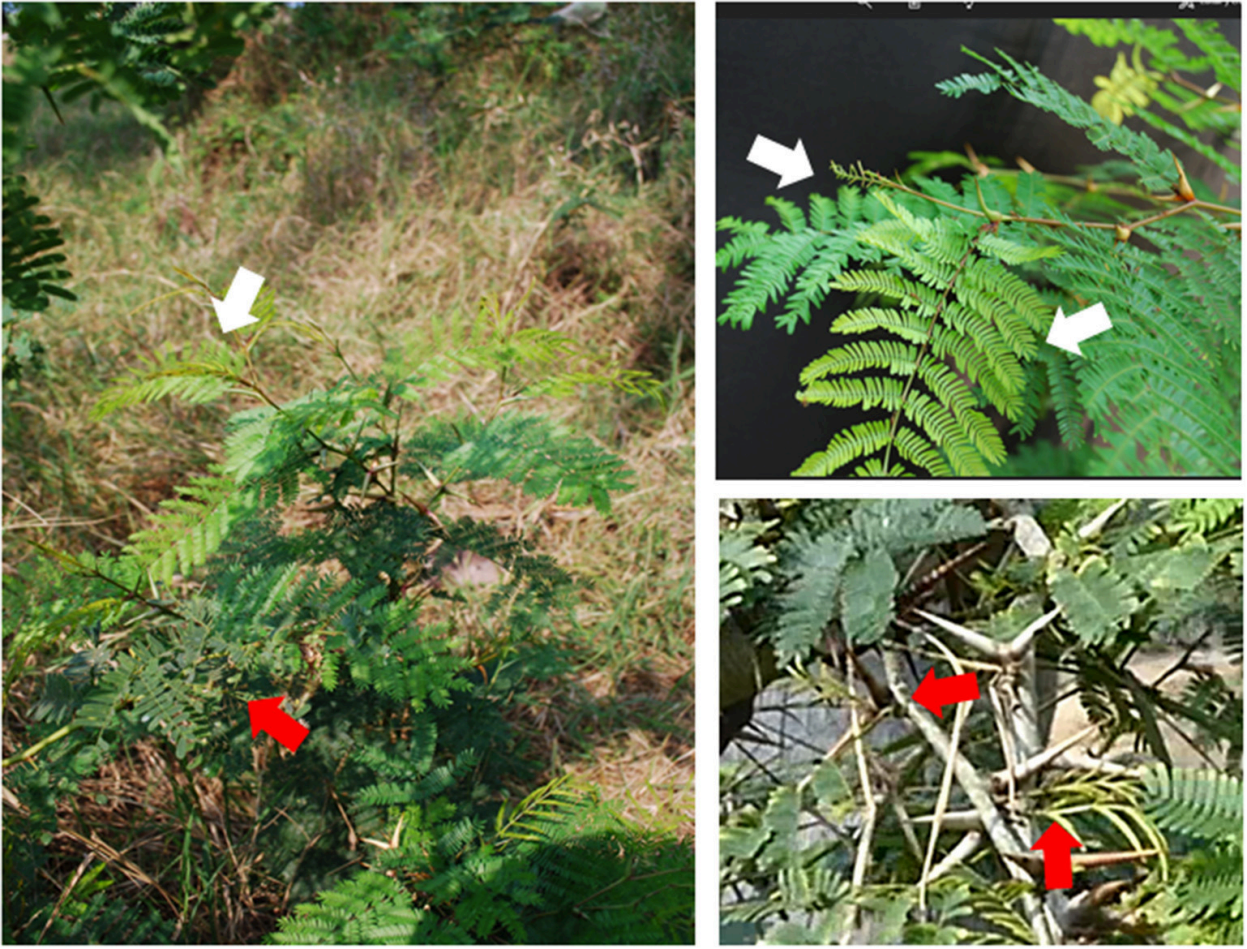
Modular colonization and defence of an *A. cornigera* plant. Obligate myrmecophytes can carry small ant colonies that protect only the colonized branches (white arrows) whereas the remaining parts of the plant remains free of ants and, thus, without a protection from herbivores and pathogens (red arrows). This photo is a part of Supplementary Video File 1. See video file for close-ups.

In the present study, we used two EFN-secreting plant species from the same genus to test the hypothesis that the obligate ant-plant provides this reward in a modular way whereas the facultative ant-plant provides the reward systemically, and to identify the molecular mechanism that allows for a modular versus systemic reward production. Obligate ant-plants such as the Mesoamerican “Swollen-thorn Acacias” (*sensu* Janzen, 1974), such as *A. cornigera, A. hindsiii* and *A. collinsii*, offer EFN and cellular food bodies as food rewards for obligate plant-ants from the *Pseudomyrmex ferrugineus* group (Janzen, 1966; Ward, 1993; Seigler and Ebinger, 1995; Ward and Branstetter, 2017). These ants colonize their host partly, or completely, and protect the colonized parts from herbivores, climbers and pathogens (Janzen, 1967, 1969; González-Teuber et al., 2014; see Figure 2, and Supplementary Video File 1). In contrast, other species such as *A. farnesiana, A. cochliacantha* and *A. macracantha* (Seigler and Ebinger, 1988) engage in facultative mutualisms with generalist ants species that patrol these plants (Bentley, 1977; Tilman, 1978; Koptur, 1992; Agrawal, 1998). In the latter three plant species, EFN secretion has already been shown to be induced by damage or the exogenous application of JA (Heil et al., 2004). Besides herbivory, mechanical damage, or treatment with JA, EFN secretion can also be induced by volatile organic compounds (VOCs), at least in lima bean (*Phaseolus lunatus*) (Kost and Heil, 2006; Heil and Silva- Bueno, 2007). In fact, VOCs have been reported as external signals that orchestrate systemic responses to local attack in diverse plant species, comprising both monocots and dicots (Frost et al., 2008; Heil and Ton, 2008; Heil and Karban, 2010; Schrader et al., 2017). Considering this role of VOCs, we hypothesized that a modular versus systemic production of an inducible reward like EFN could result from differences in the emission of plant VOCs or in the responsiveness of the plant to these VOCs (Figure 1). In short, in this study, we employ a comparative approach to investigate whether plant VOCs can act as airborne plant hormones that generate different spatial patterns in the EFN secretion by a facultative and an obligate ant-plant.

## Materials and methods

### Plant species and study site

The plant species used in this study were *Acacia cornigera* (L.) Willdenow, an obligate ant-plant, and *Acacia cochliacantha* Humb. Bonpl. ex Willd., a facultative ant-plant. All plants selected for this study were shrubs 1–2.5 m tall growing at their natural site in in the coastal area in Southern Mexico close to Puerto Escondido, Oaxaca (~15°55′N and 097°09′W). Plant species were determined following Janzen, 1974 and Seigler and Ebinger (1988, 1995), and ant species were determined following (Ward, 1993; Ward and Branstetter, 2017), and confirmed by P.S. Ward. Due to the polyphyly of the former genus *Acacia* s.l. it has been recommended to term the Mesoamerican clade of the former genus *Acacia* “*Vachellia*” (Orchard and Maslin, 2003; The Legume Phylogeny Working Group, 2017), a suggestion that has been discussed intensively (Luckow et al., 2005; Smith and Figueiredo, 2011; Kyalangalilwa et al., 2013). For the sake of reproducibility and in order to allow comparisons with published work, we respect the iconic term “Swollen thorn Acacias” as introduced by Janzen in 1974 and, hereinafter, use the species names as defined in the beforementioned taxonomic keys, which have been used to identify our study species.

### Effect of JA and ants on EFN secretion

**Figure 3:**
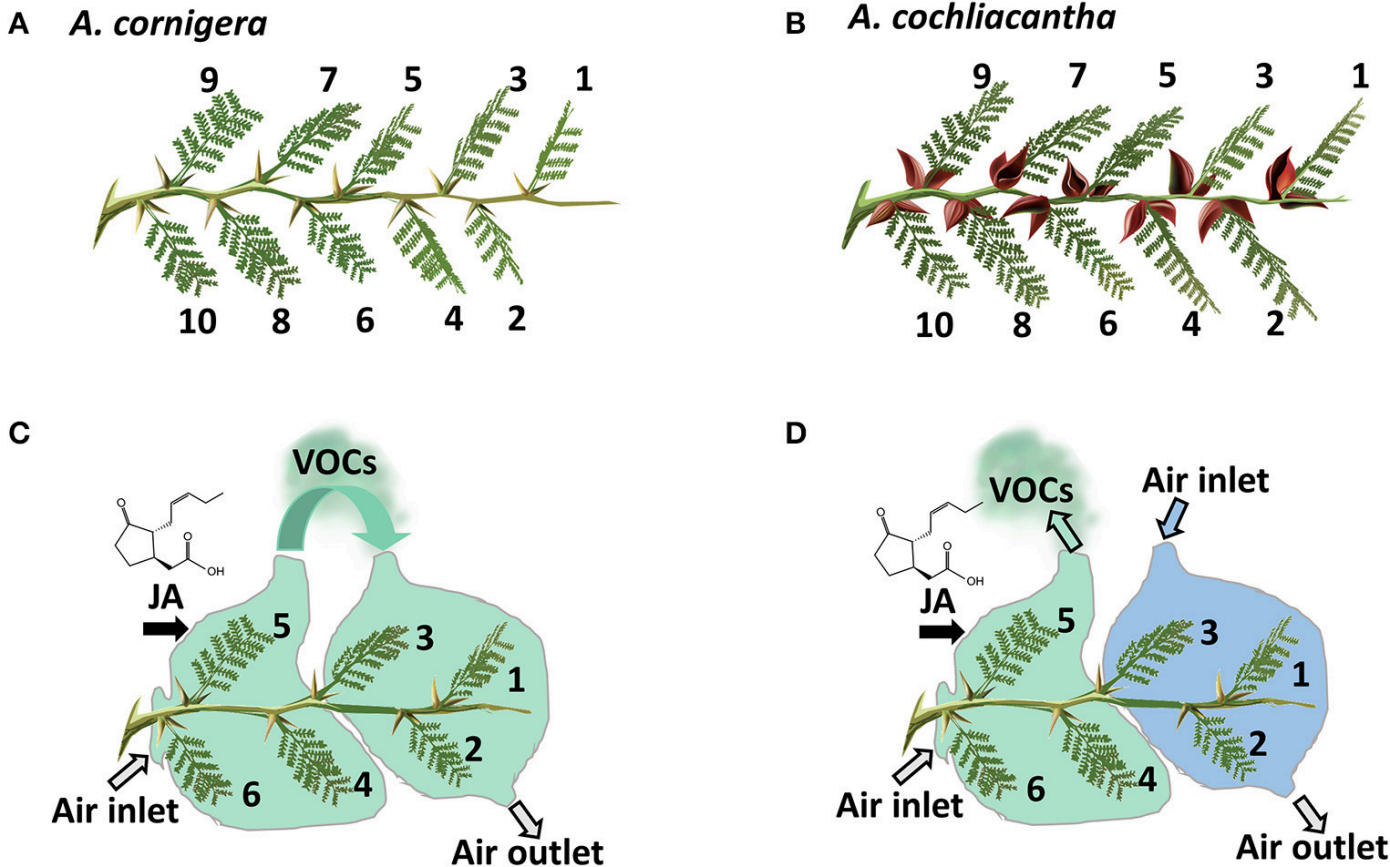
Principles of experimental design. Of both study species (**A**, obligate ant-plant: *A. cornigera*; **B**, facultative ant-plant: *A. cochliacantha*), branches used in the experiments possessed at least 10 (last experiment: six) healthy leaves. Leaves were numbered in the order of their insertion on the branch, starting with the youngest fully expanded leaf. In the experiments aimed at understanding the role of volatile organic compounds (VOCs) as systemic signals, exposure of the youngest leaves (1-3) to the VOC-containing headspace of jasmonic acid (JA)-treated mature leaves (4-6) was controlled by bagging the treated leaves in PET foil and moving the airflow toward the younger leaves **(C)** or away from the plant **(D)**. See materials and methods section for details.

Earlier observations suggested that obligate *Acacia* ant-plants secrete EFN constitutively (Heil et al., 2004). However, only plants that were colonized by the obligate plant-ant, *P. ferrugineus*, had been used in that study, which tempted us to hypothesize a role of the ants in EFN secretion. In order to investigate the effects of ants and of exogenous JA application on EFN secretion, we selected each eight plants of *A. cornigera* and of *A. cochliacantha*; all *A. cornigera* plants were inhabited naturally by *P. ferrugineus* ants, whereas the *A. cochliacantha* plants were visited by generalist ants such as *Camponotus truncatus, Crematogaster larrea, Pseudomyrmex gracilis* (species kindly determined by P.S. Ward). From each plant, we selected three branches that were similar in terms of leaf number and age of the branch, and free of visible damage. All branches possessed at least ten healthy leaves, which were numbered according to their age (leaf 1 being the youngest one, Figure 3). One branch per plant served as positive control, i.e., it remained inhabited by *P. ferrugineus* ants (*A. cornigera*) or with continuous access for generalist ants (*A. cochliacantha*). The other two branches were deprived of ants as described earlier (Heil et al., 2004). In short, all thorns were cut off (*A. cornigera* only) and all ants were removed from the branch. Then, a ring of Tangle Trap® (a sticky resin that prevents ants from passing, The Tangle Foot Company http://www.planetnatural.com/product/tree-tanglefoot-insect-barrier/) was applied around the base of the branch and finally, the branch was covered with a gauze bag to protect the EFN from flying nectar robbers and the leaves from herbivores (Heil et al., 2004). After 2 days, nectaries on one of these ant-free branches were treated with 20 μL of 1 mM aqueous solution of JA pipetted directly on each nectary or, as a negative control, with 20 μL of Milli-Q® water. Then, all three branches were deprived of ants and protected from nectar consumers as described above and in Heil et al. (2004). The volume and concentration (in equivalents of sucrose) of the secreted EFN was quantified 24 h later with microcapillaries and a portable refractometer (Atago® hand refractometer) for three leaves per branch. Consecutively, these leaves were collected and dried to express EFN secretion as amounts of soluble solids secreted per g of leaf dry mass and 24 h, as described earlier (Heil et al., 2004).

### Modular vs. systemic response to JA

In order to evaluate whether the two Acacia species investigated here secrete EFN as a modular or a systemic response, we selected eight plants each of *A. cornigera* and *A. cochliacantha* as described above and selected two branches per plant, using the same criteria as above but making sure that each branch had at least ten fully expanded, healthy leaves. One branch per plant was treated by applying 20 μL of 1 mM of aqueous JA solution to the nectaries of leaves 1, 5, and 10, whereas 20 μL of Milli-Q® water was applied to the same nectaries on the control branches. After 24 h, EFN secretion was quantified as described above. However, in this experiment, EFN secretion was quantified individually for each leaf, including both the “local” (i.e., treated) as well as the “systemic” (i.e., non-treated) leaves of the same branch.

In order to quantify CWIN activity, an independent set of eight plants from each species were selected and treated as described above. The nectary tissue was collected 1 h after JA-treatment and stored immediately on dry ice in 1.5 mL Eppendorf tubes. Samples were transferred to a portable Deep Freezer (https://www.thomassci.com/Equipment/General-Purpose-Refrigeration/_/25L-Super-Low-Temperature-Portable-Deep-Freezer?=&q=Ultra+Low+Freezer) and stored at −40°C until use. The activity of CWIN was quantified according to Millán-Cañongo et al. (2014) and Ruhlmann et al. (2010) with some modifications. Briefly, 0.10 g of frozen tissue was ground and mixed with 500 μL cold 50 mM HEPES/NaOH (pH 8.0, containing 5 mM MgCl_2_, 2 mM EDTA, 1 mM MnCl_2_ and 1 mM CaCl_2_). Samples were incubated on ice for 10 min and then centrifuged at 13,000 g for 10 min at 4°C. The pellets that contained the cell walls with associated invertases were washed three times with 500 μL extraction buffer by resuspension and centrifugation as described above, each time discarding the supernatant. Then, the pellets were washed three times with 500 μL of 80 mM sodium acetate (pH 4.8). Then, 300 μL of 80 mM sodium acetate (pH 4.8) were added to the pellets, suspended, and the mixture was incubated at 37°C. Every 5 min, an aliquot of 20 μL was taken and mixed with 200 μL of hexokinase (HK) reaction solution (“glucose (HK) assay kit”; Sigma-Aldrich, http://www.sigmaaldrich.com). After reaching steady state, 100 μL of an aqueous 100 mM solution of sucrose was added, and the absorption was measured at 340 nm in a lQuant® Spectrophotometer, ThermoSpectronic, microplate reader every 5 min for 40 min with Gen 5 software (Biotek https://www.biotek.com/products/software-robotics-software/gen5-microplate-reader-and-imager-software/).

### Effect of VOCs on EFN secretion

In order to investigate a putative role of VOCs on EFN secretion, we adapted the experimental design from (Heil and Silva- Bueno, 2007) in which the youngest leaves of a branch served as local “receiver” leaves to which air flow from the headspace of other leaves on the same branch was experimentally manipulated (Figure 3). In short, four plants each of *A. cornigera* and *A. cochliacantha* were selected as described above. Five branches that possessed at least six healthy leaves (of which the youngest leaves 1-3 served as receivers) were selected on each plant and were deprived of ants as described above. Then, the branches were subjected to one of the following treatments (see Figure 3 for details). In the first treatment (Figure 3C), three mature leaves (4-6) were treated by spraying a 1 mM aqueous JA solution until the surfaces of all leaves were covered, allowed to dry, and then bagged in plastic foil (“Bratschlauch”, Toppits, Minden, Germany; a PET foil that does not emit detectable amounts of volatiles). One side of the bag was left open and an open-flow system was created by placing a plastic tube (30 × 2 cm; inner surface lined with Bratschlauch) on the opposite side, creating a continuous air flow placing a ventilator (video card cooler “Evercool EC-4010,” Steren, Mexico City, Mexico, supplied with 4.5 V) at the upper end of the tube. Then, air flow from the treated leaves was directed toward the three youngest leaves (1-3) on the same branch. In the second treatment (Figure 3, Panel D), the air flow from the treated leaves was directed away from the branch, leaving leaves 1-3 exposed to ambient air. As a control, we manipulated air flow was as in the first treatment, but leaves 4-6 were treated with water as a control. Ultimately, two branches of the same plant were exposed to air coming either from a JA-treated or a Milli-Q® water-treated branch of the other study plant species (i.e., leaves of *A. cornigera* were exposed to air coming from *A. cochliacantha* or *vice-versa*).

### Collection and analysis of VOCs

In order to compare the VOC profiles of *A. cornigera* and *A. cochliacantha* plants, two branches of each ten plants per species were selected and the youngest 10 leaves on one branch per plant were spray-treated with 1 mM of aqueous JA solution as described above, whereas the youngest ten leaves on the other branch were spray-treated with water as a control. After allowing leaves to dry, the branches were bagged in Bratschlauch. VOCs were collected over 24 h in a closed-loop system as described in Donath and Boland (1995), using micro-pumps (model DC 06/21 FK, Fürgut, Tannheim, Germany) and filters (1.5 mg of charcoal, CLSA- Filters, Le Ruissaeu de Montbrun, France). The VOCs were desorbed from the filters using 40 μL of dichloromethane with 1- bromodecane (98%, Aldrich) at a concentration of 100 ng μL^−1^ as an internal standard, and samples were injected directly into a gas chromatograph-electron impact ionization mass spectrometer (GC- EIMS) system (Agilent 7890 series gas chromatograph interfaced to an Agilent 5975 electron impact ionization mass-selective triple axis detector; Agilent Technologies Santa Clara, CA, USA). The separation was performed using a HP5- FAPP column (30 m long, 0.32 mm internal diameter and 0.5 mm film thickness) under the following conditions. Injector temperature 180°C, detector temperature 230°C, initial temperature 70°C, then ramped up at 5°C min^−1^ to 120°C, then ramped at 8°C min^−1^ to a final temperature of 210°C, which was maintained for 12 min. The mass spectra were analyzed with MassHunter 2017 by Agilent Technologies®, and compounds were preliminarily annotated with “NIST MS Search Program v.2.0g,” Library version 11, and AMDIS version 2.71 from Agilent Technologies®, and then confirmed with authentic standards purchased from Sigma-Aldrich and Fluka Chemie (now Merck, purchased via Sigma-Aldrich, Toluca, Mexico).

### Statistical analysis

The all data obtained were subjected to *t-s*tudent tests or ANOVA with posthoc Tukey-HSD. For the statistical analyses we used the program R® version 3.3.0 (R studio).

## Results

### Effects of JA on EFN secretion and CWIN activity

**Figure 4:**
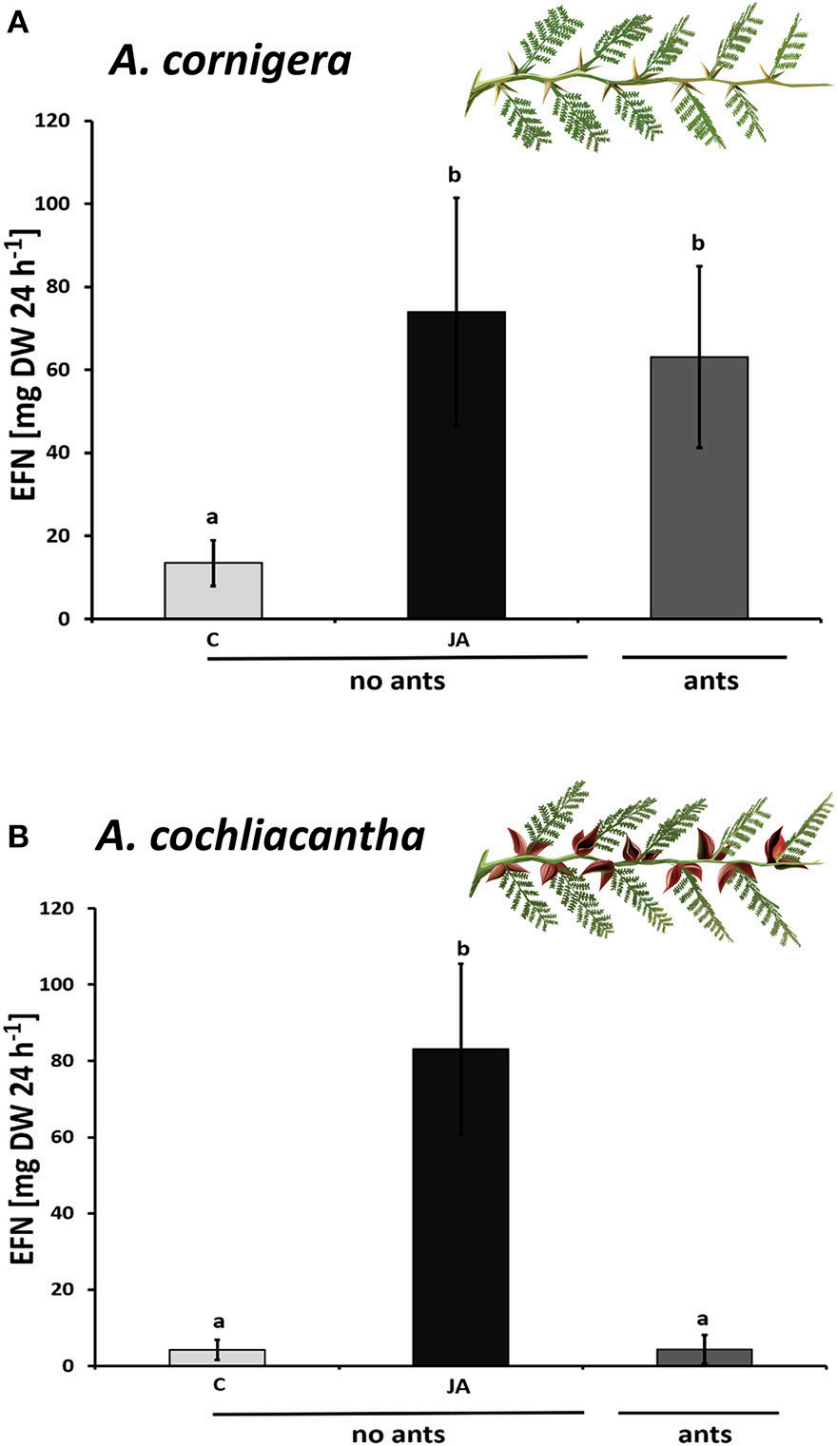
Exogenous JA induces EFN secretion in *A. cornigera* and *A. cochliacantha*. Extrafloral nectar (EFN) secretion on ant-free branches of **(A)** *A. cornigera* and **(B)** *A. cochliacantha* responded to exogenous JA (black bars) as compared to controls (light gray bars). EFN secretion was also high on *A. cornigera* branches that were colonized by mutualistic (*P. ferrugineus*) ants but not on *A. cochliacantha* branches that were visited by generalist ants (dark gray bars). Bars represent means ± SD of EFN secretion rates in mg of sucrose equivalents per g of leaf dry mass and 24 h, different letters above bars indicate significant differences (*p* < 0.001, according to ANOVA followed by Tukey HSD, *n* = 8).

**Figure 5:**
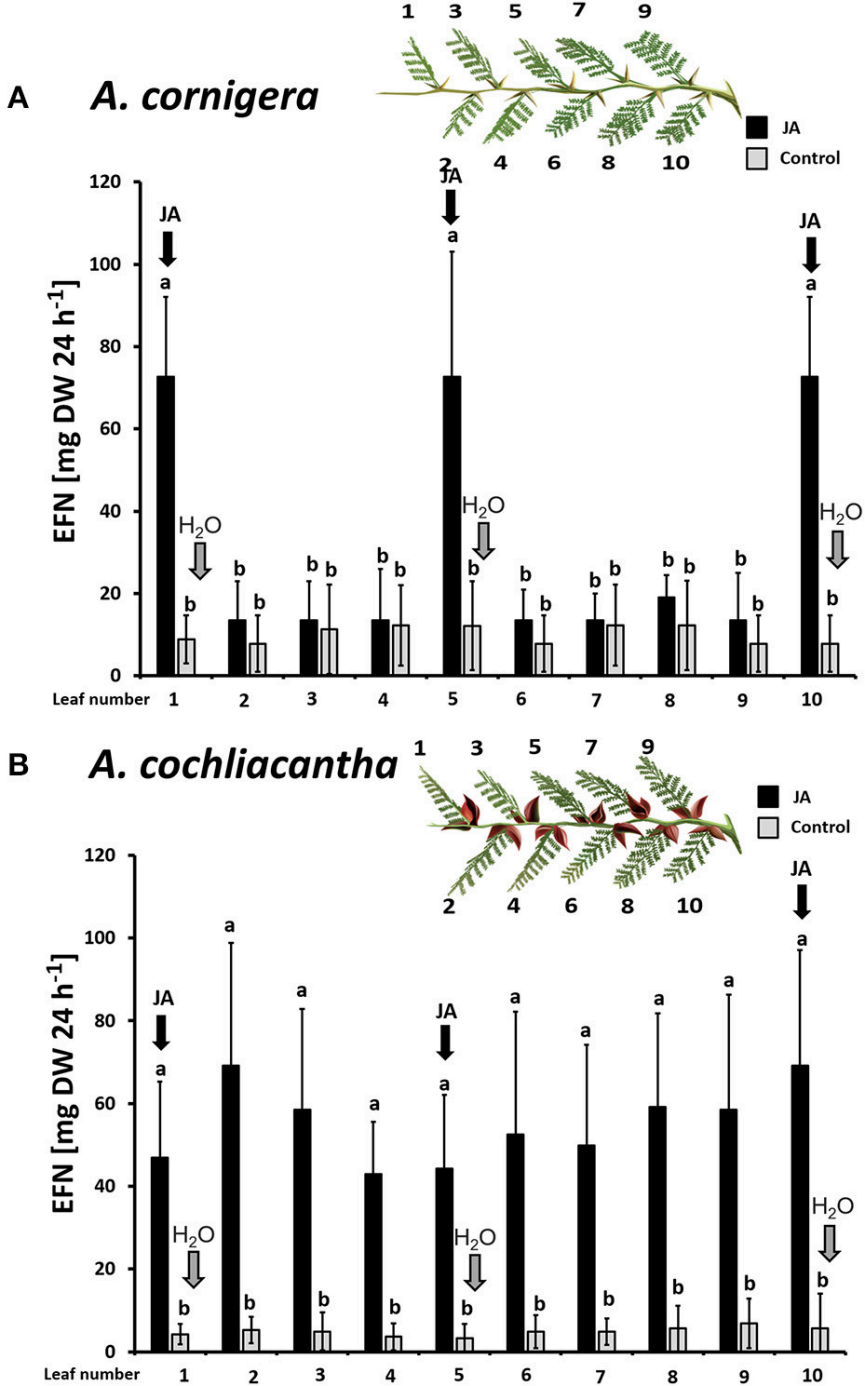
Exogenous JA induces EFN secretion locally in *A. cornigera* but systemically in *A. cochliacantha*. Jasmonic acid (JA) was applied to leaves 1, 5, and 10 on ant-free branches of *A. cornigera* **(A)** or *A. cochliacantha* **(B)** and extrafloral nectar (EFN) secretion was quantified individually on all 10 leaves of these treated branches (black bars). Control branches (gray bars) received Milli-Q® water on leaves 1,5 and 10. Bars represent means ± SD of EFN secretion rates in mg of sucrose equivalents per g of leaf dry mass and 24 h different letters above bars indicate significant differences (*p* < 0.001, according to ANOVA followed by Tukey HSD, *n* = 8).

Plants of *A. cornigera* secreted significantly more EFN on ant-inhabited and JA-treated ant-free branches as compared to water-treated ant-free branches (*p* < 0.001 for the comparisons ants *vs*. ant-free and JA *vs*. ant-free; *p* > 0.05 for ants *vs*. JA; see Figure 4A). In contrast, in the case of *A. cochliacantha*, only JA treatment had a significant effect on EFN secretion (p < 0.001; see Figure 4B), whereas no significant difference could be detected between ant-free branches and branches to which ants had access (*p* > 0.05, see Figure 4B). The differences between both species became even more pronounced when we investigated the systemic effects of local JA application (Figure 5). In the case of *A. cornigera*, application of JA to the nectaries on leaves 1, 5, and 10 significantly induced EFN secretion in the directly treated nectaries (difference between treated and control leaves: *p* < 0.001 at leaf positions 1, 5, and 10), whereas the EFN secretion rates on the systemic leaves of the treated branches showed no significant difference to the secretion rates on the corresponding leaves on untreated branches (*p* > 0.05 at leaf positions 2, 3, 4, 6, 7, 8, and 9; see Figure 5A). In contrast, all leaves on the treated branches of *A. cochliacantha* responded with a significant increase in EFN secretion to JA application to the nectaries on leaves 1, 5, and 10, independently whether they were “local” (i.e., directly treated) or “systemic” leaves (difference between leaves on treated and control branches: *p* < 0.001 at all 10 leaf positions, see Figure 5B).

**Figure 6:**
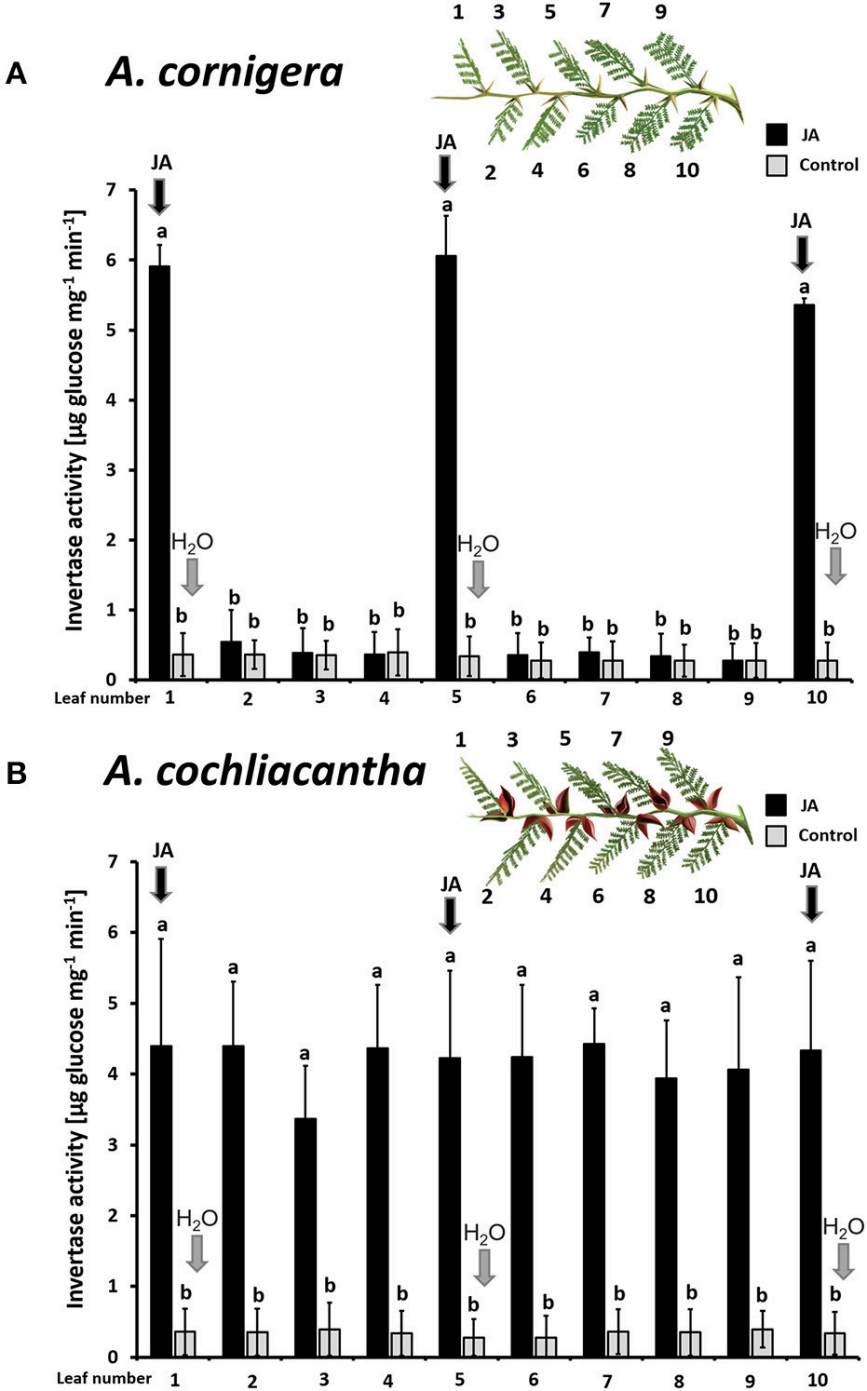
Exogenous JA induces invertase activity in nectary tissue locally in *A. cornigera* but systemically in *A. cochliacantha*. Jasmonic acid (JA) was applied to leaves 1, 5, and 10 on ant-free branches of *A. cornigera* **(A)** or *A. cochliacantha* **(B)** and invertase activity was quantified individually on all 10 leaves of these treated branches (black bars). Control branches (gray bars) received Milli-Q® on leaves 1, 5, and 10. Bars represent means ± SD of invertase activity in μg of glucose per mg of nectary tissue per min, different letters above bars indicate significant differences (*p* < 0.001 according to ANOVA followed by Tukey HSD, *n* = 8).

The same patterns were observed in CWIN activity. In *A. cornigera*, CWIN activity in nectary tissue responded significantly (*p* < 0.001) to the direct JA application to nectaries on leaves 1, 5, and 10, but showed no significant differences between nectaries on leaves 2, 3, 4, 6, 7, 8, and 9 on JA-treated versus control branches (*p* > 0.05) (Figure 6A). In the case of *A. cochliacantha*, however, CWIN activity was significantly higher in all leaves on the treated branches as compared to the corresponding leaves on control branches (*p* < 0.001 at all 10 leaf positions), and no differences could be detected among the “local” and the “systemic” leaves on the treated branches (Figure 6B).

### VOCs from *A. cochliacantha* induce EFN secretion in both species

**Figure 7:**
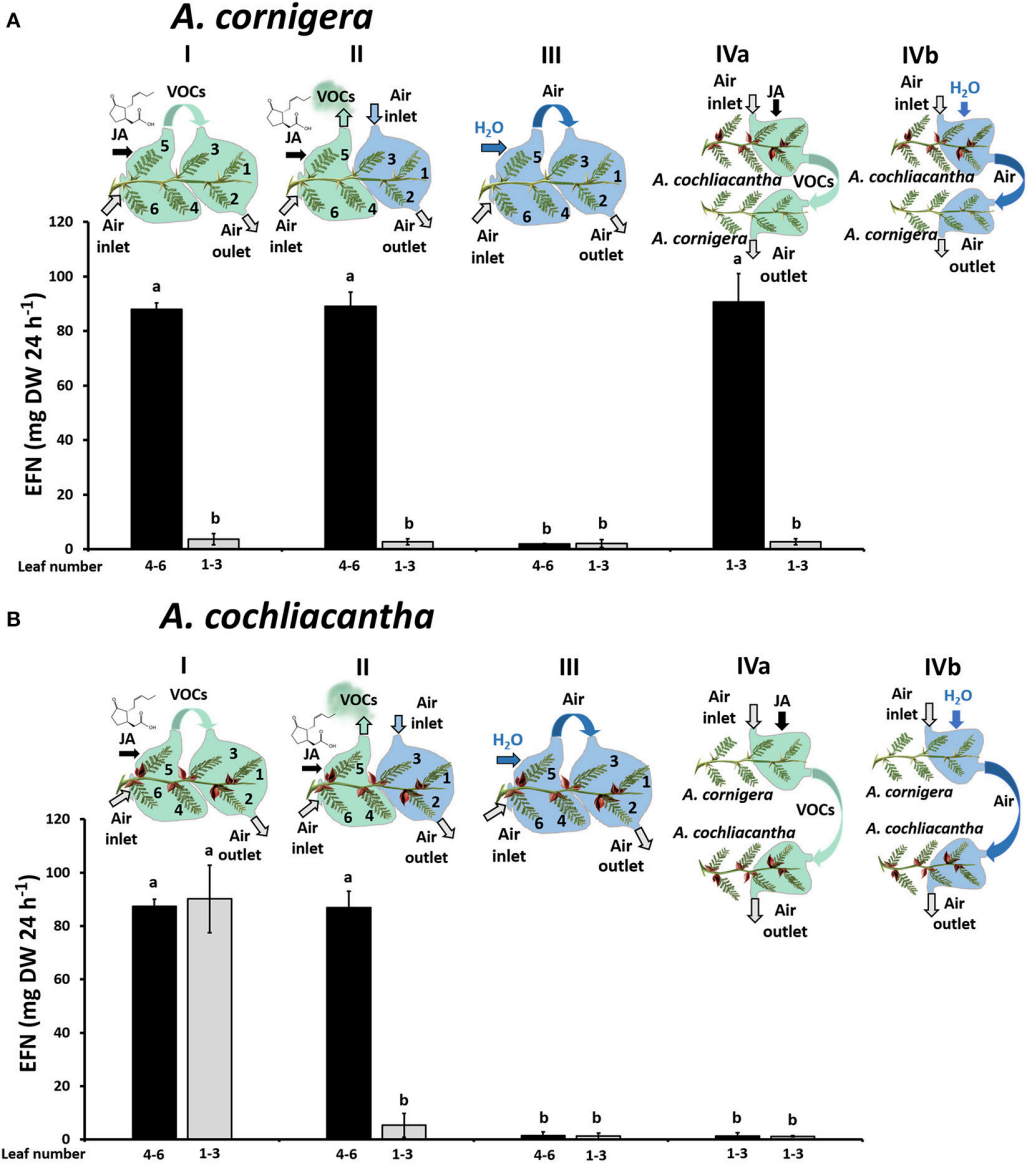
The headspace of JA-treated *A. cochliacantha* branches induces extrafloral nectar (EFN) secretion in both plant species. The secretion of EFN on ant-free branches of *A. cornigera* **(A)** and *A. cochliacantha* **(B)** branches is depicted for young leaves (no. 1–3) and mature leaves (4–6) that were treated with JA or exposed to the headspace of differently treated leaves. Treatment I, mature leaves treated with JA, young leaves exposed to air from mature (i.e., induced) leaves. Treatment II, mature leaves treated with JA, young leaves exposed to environmental air. Treatment III, mature leaves treated with Milli-Q® water, young leaves exposed to air from mature (i.e., non-induced) leaves. Treatment IV, response of young leaves (1–3) to the headspace from heterospecific leaves (i.e., *A. cornigera* exposed to headspace of *A. cochliacantha* and vice-versa). The heterospecific emitter branches were either treated with JA (IVa) or with Milli-Q® water (control, IVb). Bars represent means ± SD of EFN secretion rates in μg per g leaf dry mass and 24 h, different letters above bars indicate significant differences (*p* < 0.001 according to ANOVA followed by Tukey HSD, *n* = 4).

When we treated mature leaves (4-6) of *A. cornigera* with JA to study the putative role of JA-responsive VOCs, the EFN secretion by these leaves was induced, as shown by the significant differences (*p* < 0.001) between EFN secretion rates observed on JA-treated leaves versus controls (Figure 7A). In contrast, the EFN secretion on the young leaves (1–3) of the same branches did not respond significantly to exogenous JA applied to the mature leaves, independently of whether the young leaves were exposed to the headspace of the treated, mature leaves (*p* < 0.05) or not (Figure 7A, treatments I and II). However, EFN secretion on young leaves (1–3) of *A. cornigera* was significantly induced after the exposure to the headspace of JA-treated leaves of *A. cochliacantha* (*p* < 0.001, see Figure 7A, treatment IVa). Correspondingly, EFN secretion on mature leaves of *A. cochliacantha* responded significantly to exogenous JA (Figure 7B), and the EFN secretion on young leaves was induced by the headspace of JA-treated mature leaves: that is, EFN secretion on leaves 1-3 was significantly higher (p < 0.001) on leaves that had been exposed to the headspace coming from JA-treated mature leaves (Figure 7B, treatment I) as compared to ambient air or the headspace of water-treated control leaves (Figure 7B, treatments II and III). Finally, no significant effect (*p* > 0.05) on EFN secretion by *A. cochliacantha* could be detected for the headspace of *A. cornigera*, independently of whether the *A. cornigera* branch had been treated with JA or not (Figure 7B, treatment IVa,b).

### The voc blends of *A. cornigera* and *A. cochliacantha* are different

**Figure 8:**
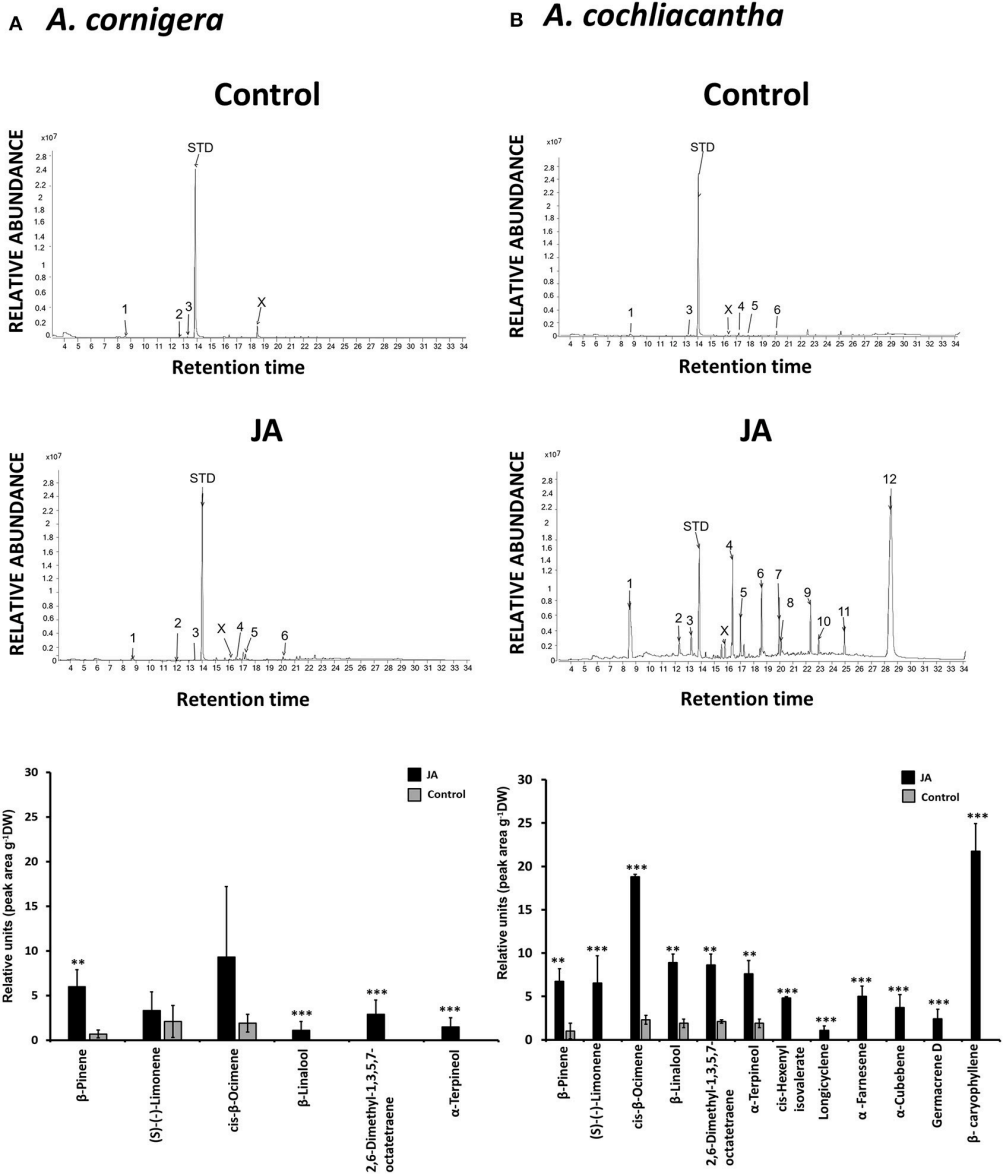
Volatile organic compounds (VOCs) in the headspace of both Acacia species. Representative GC-spectra of VOCs are depicted for **(A)** *A. cornigera* and **(B)** *A. cochliacantha* branches treated with Milli-Q® water (control) or jasmonic acid (JA). Bar charts present average peak areas (× 107) per g of dry leaf mass for *n* = 10 independent samples per species and treatment. Asterisks indicate the results of *t*-tests comparing control vs. JA-treatment (^***^P < 0.001, ^**^P < 0.005).

Both species responded to exogenous JA with the induced emission of various VOCs (Table 1). We could detect six different compounds in the headspace of JA-treated *A. cornigera* branches, among which the monoterpene alcohols, α-terpineol and β-linalool, and 2,6-dimethyl-1,3,5,7-octatetraene (“dimethyl-octatetraene” in Figure 8A), were released only from JA-treated plants (Figure 8A). All six compounds that we detected in the headspace of JA-treated *A. cornigera* branches could also be detected in the headspace of JA-treated *A. cochliacantha* branches. However, *A. cochliacantha* emitted six additional compounds: *cis*-hexenyl isovalerate, the monoterpene, α-cubebene, and the sesquiterpenes longicyclene, germacrene, β-caryophyllene and α-farnesene (Figure 8B). According to t-tests performed separately for each VOC, JA treatment induced the emission of four out of six compounds significantly (p < 0.005) in case of *A. cornigera* and of all 12 compounds in case of *A. cochliacantha* (Table 1, Figures 8A,B).

**Table 1:** Volatile organic compounds (VOCs) detected in the headspace of *A. cornigera* and *A. cochliacantha*.

			***A. cornigera***		***A. cochliacantha***	
**Compound**	**Peak**	**RT**	**JA**	**Control**	**JA**	**Control**
β-Pinene	1	7.18	6.0 ± 1.9	0.7 ± 0.4	6.7 ± 1.4	1.0 ± 09
(S)-(-)-Limonene	2	8.46	3.3 ± 2.1	2.1 ± 1.8	6.5 ± 3.1	ND
*cis*-β-Ocimene	3	13.15	9.3 ± 7.9	1.9 ± 1.2	18.8 ± 0.2	2.3 ± 0.5
β-Linalool	4	16.18	1.1 ± 1.0	ND	8.9 ± 0.9	1.9 ± 0.5
2,6-Dimethyl-1,3,5,7-octatetraene	5	17.29	2.9 ± 1.6	ND	8.6 ± 1.2	2.1 ± 0.2
α-Terpineol	6	18.32	1.5 ± 1.0	ND	7.6 ± 1.5	1.9 ± 0.5
*cis*-Hexenyl isovalerate	7	19.53	ND	ND	4.8 ± 0.1	ND
Longicyclene	8	21.27	ND	ND	1.1 ± 0.5	ND
α-Farnesene	9	23.28	ND	ND	5.1 ± 1.2	ND
α-Cubebene	10	24.64	ND	ND	3.7 ± 1.5	ND
Germacrene D	11	26.02	ND	ND	2.4 ± 1.1	ND
β-Caryophyllene	12	28.29	ND	ND	21.7 ± 3.2	ND

*The headspace was sampled over 24 h after the treatment and compounds identified in Figure 8 as numbered peaks are listed according to their retention time (RT), values represent mean peak areas (× 10^7^) ± SD per g of dry mass of the emitting leaves of n = 10 independent samples per species and treatment, ND, Not detected. All compounds were confirmed by co-injection with commercial standards*.

## Discussion

In the present study, we show that EFN secretion by the obligate ant-plant, *A. cornigera*, responded in a modular manner to local JA-treatment, whereas in the facultative ant-plant, *A. cochliacantha*, EFN secretion responded more systemically. The activity of CWIN in the tissue of the individual nectaries closely resembled the patterns seen in EFN secretion rates in both species, confirming earlier reports that EFN secretion is controlled at the level of individual nectaries (Orona-Tamayo et al., 2013). In *A. cornigera*, an enhanced EFN secretion also correlated with the colonization by resident mutualistic *P. ferrugineus* ants, whereas generalist ants visiting the nectaries of *A. cochliacantha* did not exert any detectable EFN-inducing effect. A modular sanctioning of non-reciprocating symbionts has been reported from different types of mutualisms. However, the signals that allow for different spatial patterns in the production of rewards remain to be identified. Thus, the observation of different spatial patterns in the reward production by two closely related species made our system highly suitable to search for the causal mechanism that controls these patterns.

### EFN secretion in both species responds to *A. cochliacantha* VOCs

Interestingly, the headspace of JA-treated leaves of *A. cochliacantha* induced EFN secretion in the systemic leaves of the same branch. As reported earlier, inducible VOCs can act as airborne “external” signals that control systemic responses to local damage (Heil and Ton, 2008; Scala et al., 2013; Loreto et al., 2014). In contrast, EFN secretion by systemic leaves of *A. cornigera* did not respond to the headspace of the JA-treated leaves of the same branch. This observation shows that in *A. cornigera*, VOCs do not serve as a systemic, EFN-inducing signal, and raised the question whether the two species studied here differ in the emission of VOCs, or in the responsiveness of EFN secretion to these VOCs. Indeed, the headspace of JA-induced *A. cochliacantha* branches readily induced EFN secretion in *A. cornigera*. Although an exposure of *A. cornigera* leaves to VOCs from *A. cochliacantha* is not likely to resemble a natural situation of ecological relevance, this finding clearly demonstrates that EFN secretion by *A. cornigera*, in principle, can be induced by exogenous volatile signals.

### VOCs as plant-to-ant signals

Which ones among the VOCs that were emitted from *A. cochliacantha* were responsible for the EFN-inducing effect? The role of VOCs in the signaling from host plants to their ants has been studied in various ant-plant systems. In *Acacia* spp. and *Macaranga* spp., VOCs emitted from facultative and obligate ant-plants were compared to identify compounds that might serve as host-finding cues for foundresses (Jurgens et al., 2006; Razo-Belman et al., 2018). Agrawal (1998) was the first to focus on the defensive aspect and studied plant-ants as a VOC-responsive mechanism of protection. Inui and Itioka (2007) found that diverse species of *Macaranga* myrmecophytes emitted different VOC profiles when damaged and that the aggressiveness of resident *Crematogaster* ants toward damaged leaves also differed among the host species. Using leaf pieces from different *Piper* species, Mayer et al. (2008) found that the resident ants responded more strongly to damaged leaves from obligate than from facultative *Piper* ant-plants, whereas Schatz et al. (2009) used the same approach to show that obligate plant-ants responded to leaf pieces of their host plant more strongly than a non-defending exploiter ant (Schatz et al., 2009). Curiously, this behavior could be elicited using, among other VOCs, pure hexanal (Schatz et al., 2009), a green leaf volatile already reported by Agrawal (1998) to elicit practically the same response as plant sap in *Azteka* plant-ants. That is, ubiquitous plant VOCs that are emitted from most plants upon damage, or even infection (Heil, 2014; Quintana-Rodriguez et al., 2015, 2018), might be the triggers that ants use to detect host plant damage. More importantly, all the beforementioned six studies focused on the direct chemical communication from the plant to the ants, whereas in the present study, we focused on the VOC-mediated signaling within the plant.

### Plant VOCs as defence-inducing hormones

The headspace of *A. cornigera* caused no detectable induction of EFN secretion, which makes it reasonable to assume that the active VOCs can be found under those compounds that were emitted only, or in much higher amounts, by *A. cochliacantha*. Defence-inducing effects are commonly reported for green leaf volatiles such as (*E*)-2-hexenal or (Z)-3-hexenyl acetate (Scala et al., 2013; von Mérey et al., 2013; Loreto et al., 2014; Sharma et al., 2017). For example, *cis*-hexenyl isovalerate has been reported to induce EFN secretion in *Phaseolus lunatus* (Heil et al., 2008) and indeed, this compound could be detected only in the headspace of JA-treated *A. cochliacantha* branches. We could not detect other green leaf volatiles in our analyses, which might be partly due to the particular difficulties to collect small, highly volatile compounds under field conditions or to the detection threshold of our GC-MS analyses. Nevertheless, our control samples were practically free of detectable VOCs and all six VOCs that we detected in the headspace of *A. cornigera* had already been reported from this plant species (Razo-Belman et al., 2018). These facts make us confident that our results adequately resemble the major VOCs that are emitted from our study species. Five of the six compounds that were exclusive for *A. cochliacantha* were mono- or sesquiterpenes, a group of VOCs for which defense-inducing effects are much less frequently reported than for green leaf volatiles (Sharma et al., 2017). However, an artificial blend consisting of *R*- (–)-linalool, β-caryophyllene, methyl salicylate, *cis*-jasmone, (*cis*)-3-hexenyl acetate, β-ocimene, (3*E*)-4,8-dimethylnona-1,3,7-triene (DMNT) and (3*E*,7*E*)-4,8,12-trimethyltrideca-1,3,7,11-tetraene (TMTT) induced EFN secretion in lima bean (*Phaseolus lunatus*) (Kost and Heil, 2006), DMNT and TMTT induced pathogenesis-related (PR) genes and lipoxygenase (a central step in the synthesis of JA) in *P. lunatus*, and β-ocimene induced PR genes in the same species (Arimura et al., 2000). Likewise, a mixture of α- and β-pinene induced PR1 gene expression in *Arabidopsis* (Riedlmeier et al., 2017), whereas β-caryophyllene triggered membrane depolarization, which is a very early step in plant defense induction, in *Solanum lycopersicon* (Zebelo et al., 2012). Among these compounds, β-caryophyllene and β-ocimene were the quantitatively dominant compounds in the headspace of JA-treated *A. cochliacantha* branches, and β-caryophyllene was exclusive to this species. Taken together, our results make it highly likely that VOCs that are emitted only – or in much higher amounts–from *A. cochliacantha* leaves function as a systemic EFN-inducing signal, and that the strictly modular response in EFN secretion that we observed in *A cornigera* is caused by a reduced emission of these volatile signals, rather than a reduced responsiveness to the signals.

### Optimized rewarding by modular vs. systemic responses

Modular responses in plant defense have been suggested to be driven by herbivores in order to optimize host sharing (Lee et al., 2017). However, in the case of our study system, it appears more likely that local EFN secretion enables *A. cornigera* to focus reward production on specific parts of the plant surface. EFN is a costly reward (Escalante-Pérez and Heil, 2012) and can be a limiting factor for ant colony growth (Byk and Del-Claro, 2011). In the case of Swollen-thorn Acacias, higher EFN secretion rates can shift the competitive balance between defending mutualist ants and non-defending exploiters to the benefit of the mutualists (Heil, 2013). The reduced emission of VOCs by *A. cornigera* is likely to represent a consequence of the frequently proposed reduction of direct defense traits in obligate ant-plants (Janzen, 1966; Rehr et al., 1973; Koricheva and Romero, 2012), rather than a specific adaptation to avoid a systemic induction of EFN secretion.

We also hypothesized that systemic EFN secretion enables a facultative ant- plant to attract more ants and gain a better defensive service when it is required. Although being reasonable (Agrawal and Rutter, 1998), surprisingly little evidence has been reported to support this assumption. In fact, the defensive effects of EFN secretion are highly context-dependent (Heil, 2015; Jones et al., 2017) and EFN secretion can even be counterproductive if ants start to exclude other, more efficient defenders Koptur et al., 2015). Inducing EFN secretion with exogenous JA increased the number of defending ants and decreased the number of herbivores showing up on *Macaranga tanarius* plants (Heil et al., 2001), and similar patterns were found on *P. lunatus* tendrils that were exposed to VOCs or treated with JA to enhance EFN secretion (Kost and Heil, 2008). However, all leaves had been treated in these studies, making a separation of local and systemic effects impossible. A study at the ecosystem level showed that ant abundance increased with higher EFN secretion rates and presented evidence for a strong competition among the ants for this valuable food reward (Lange et al., 2017). That ants compete for EFN has been reported from different systems (Blüthgen and Fiedler, 2004; Xu and Chen, 2010; Lange et al., 2017), which makes it likely that, under most circumstances, enhanced amounts of EFN that are secreted on larger areas of a facultative ant- plant should enhance the number of attracted ants (Figure 1).

## Conclusions

In the case of an obligate ant-plant, a modular rewarding of resident ants (Figure 2, and Supplementary Video 1) should allow for an optimized investment in protection, because these ants defend only a restricted part of the plant. In contrast, the protection of a facultative ant-plant should increase when higher numbers of visiting ants are recruited via an enhanced investment in reward production. Interestingly, volatile signals represent a molecular mechanism that controls systemic responses to local events and that can generate marked differences in the provisioning of rewards among closely related plant species that engage in different types of food-for-protection mutualisms.

## Author contributions

OH-Z and RR-B designed and performed the field experiments, analyzed the data and wrote a first version of the manuscript. MH helped with the design of the experiments and data analyses and all authors worked on the final version of the manuscript.

### Conflict of interest statement

The authors declare that the research was conducted in the absence of any commercial or financial relationships that could be construed as a potential conflict of interest.
